# Effect of heart rate on poor outcome in stroke patients treated with intra-arterial thrombectomy

**DOI:** 10.1186/s12883-024-03662-8

**Published:** 2024-05-21

**Authors:** Huaishun Wang, Longdong Xu, Li Dong, Yingzi Li, Huihui Liu, Guodong Xiao

**Affiliations:** 1 Second Affiliated Hospital of Soochow University, Suzhou, 215004 China; 2 fifth People’s Hospital of Changshu, Suzhou, 215004 China

**Keywords:** Heart rate, Variability, Thrombectomy, Stroke, Outcome

## Abstract

**Background and purpose:**

The relationship between heart rate and the prognosis of patients with large vessel occlusion strokes treated with mechanical thrombectomy (MT) is not well established. This study aimed to evaluate the association of mean heart rate and heart rate variability (HRV) with the clinical outcomes after MT therapy.

**Methods:**

Acute ischemic stroke patients undergoing MT therapy were prospectively recruited from March 2020 to November 2022. Their heart rate was collected every hour for the initial 72 h after MT procedure, and the variability of heart rate was measured by standard deviation (SD) and coefficient of variation (CV). All-cause mortality and worsening of functional outcome (change in modified Rankin Scale (mRS) score) at 3-month were captured. Binary logistic regression was used to evaluate the association between heart rate indicators and all-cause mortality. Ordinal logistic regression was used to evaluate the association between heart rate indicators and worsening of functional outcome.

**Results:**

Among 191 MT-treated patients, 51(26.7%) patients died at 3-month after stroke. Increased mean heart rate per 10-bpm, heart rate SD and CV per 5-unit were all associated with the increased risk of mortality (adjusted hazard ratio [aHR] with 95% CI: 1.29 [1.09–1.51], 1.19 [1.07–1.32], 1.14 [1.03–1.27]; respectively). Patients in the highest tertile of heart rate SD had an increased risk of mortality (4.62, 1.70-12.52). After using mRS as a continuous variable, we found increased mean heart rate per 10-bpm, heart rate SD and CV per 5-unit were associated with the worsening of functional outcome (adjusted odds ratio [aOR] with 95% CI: 1.35 [1.11–1.64], 1.27 [1.05–1.53], 1.19 [1.02–1.40]; respectively). A linear relationship was observed between mean heart rate or heart rate SD and mortality; while all of the heart rate measures in this study showed a linear relationship with the worsening of functional outcome.

**Conclusions:**

Higher mean heart rate and HRV were associated with the increased risk of 3-month all-cause mortality and worse functional outcome after MT therapy for AIS patients.

**Supplementary Information:**

The online version contains supplementary material available at 10.1186/s12883-024-03662-8.

## Introduction

Autonomic nervous function is often impaired during a stroke, particularly in a large stroke, and associated with worsening of functional outcome and increased risk of mortality [1, 2]. Heart rate and heart rate variability (HRV), accurately representing the balance between the sympathetic and parasympathetic nervous systems, can reflect the overall stress acting on the body, and are easy to record [3–6]. Previous study have showed that abnormal admission heart rate was associated with worse outcomes after ischemic stroke onset [7, 8]. Furthermore, other studies also provided the evidence that the mean heart rate was related to the worsening of outcomes [9, 10]. HRV could predict the risk of presence of myocardial ischemia in patients without known coronary artery disease [11] or poor outcome in those with cardiovascular disease [12]. In stroke patients, several studies demonstrated that reduced HRV was associated with the adverse outcomes after ischemic stroke [2, 13]. However, one study failed to demonstrate such relationship [10].

Although mechanical thrombectomy (MT) is an effective and guideline recommended therapy for patients with acute ischemic patients (AIS) from a large vessel occlusion (LVO), about 30–40% patients have the poor functional outcome or death [14, 15]. Several risk factors, such as the age, stroke severity, timing recanalization, the collateral status, blood pressure or blood pressure variability, etc. are known have an adverse effect on their outcome [16–19]. However, evidence on the impact of mean heart rate or variability, as direct indicators of autonomic nervous system dysfunction, on the outcome in patients with large vessel strokes has not been verified after MT therapy.

The purpose of this study was to explore the association between heart rate measurements (including mean heat rate and HRV) within 72 h after MT therapy and the14-day and 3-month outcome (all-cause mortality and functional outcome).

## Methods

### Participants

This study is a retrospective analysis of prospectively collected data. We analyzed data of AIS patients with large cerebral artery occlusion who underwent MT therapy in the Second Affiliated Hospital of Soochow University from March 2020 to November 2022. The inclusion criteria for this study were: (1) age ≥ 18 years old; (2) an acute cerebral ischemia confirmed by brain imaging (magnetic resonance imaging or computed tomography scan); (3) confirmed LVO by digital subtraction angiography (DSA) or computed tomography angiography (CTA) of these arteries: M1 or M2 of middle cerebral artery (MCA), A1 or A2 of anterior cerebral artery (ACA), internal carotid artery (ICA), vertebral-basilar artery, or posterior cerebral artery; (4) the procedure was conducted within 6 h from the symptom onset or between 6 and 24 h if perfusion mismatch was present. Thrombolysis was allowed before MT in patients within 4.5-hour of time widow. The exclusion criteria included: (1) without the complete 72 h heart rate records or baseline information; (2) patients died within 72 h after MT procedure; (3) missing the 3-month follow-up visit. The study protocol was approved by the Ethics Committee of the Second Affiliated Hospital of Soochow University. Written consent was obtained from all participants or their surrogate before enrollment. After excluding 23 patients who were lost to follow-up or with incomplete baseline information or 72 h heart rate records, 191 patients were entered into the final analysis of this study. The informed consent to participate was obtained from all participants and written informed consent in accordance with the Declaration of Helsinki.

### Data collection and assessment of heart rate measures

The baseline information included demographic information, medical history, clinical features, time from onset to vessel recanalization, and imaging features. Medical history included history of hypertension, diabetes mellitus, prior stroke, atrial fibrillation (AF), current smoking, drinking, heart rate lowering treatment during the first 72 h and mean SBP 72 h after MT. Clinical features included blood pressure and heart rate profile on admission, baseline National Institutes of Health Stroke Scale (NIHSS) score, low density lipoprotein cholesterol (LDL-C), high density lipoprotein cholesterol (HDL-C), Nt-pro BNP level and tPA administered. Imaging features included the site of the occluded brain artery (ICA with or without MCA/ACA) isolated MCA or ACA, and vertebrobasilar or other location), the collateral status before procedure and reperfusion status after procedure. Collateral status was assessed using the American Society of Interventional and Therapeutic Neuroradiology/Society of Interventional Radiology (ASITN/SIR) grading by DSA [20]. The etiologic subtypes of stroke were defined according to the Trial of ORG 10,172 in acute stroke treatment (TOAST) [21]. The cerebral tissue reperfusion was evaluated by modified Thrombolysis in Cerebral Infarction (mTICI) scale and classified as no perfusion (grade 0), minimal perfusion (Grade 1), partial perfusion (Grade 2: a < 2⁄3 of the entire vascular territory; b complete filling but slowly) and complete perfusion. The state of grade 2b/3 was generally regarded as successful reperfusion [22]. 

All patients were routinely admitted to the intensive care unit for at least 72 h after MT. And all patients were required to be bedridden. Heart rate of patients was routinely monitored by electrocardiograph monitor after MT procedure during hospitalization in stroke units or neuro critical care units and entered into the electronic medical records. We did not conduct R-R interval analysis and that we only used heart rate measured at specific hours. We acquired the initial 72 h of hourly heart rate information after MT therapy and calculated HRV using 2 statistical methodologies, i.e. standard deviation (SD) and coefficient of variation (CV). The approach we take is the same as that of other articles [23, 24].

$$\text{S}\text{D}=\sqrt{\left(\frac{1}{n-1}\right){\sum }_{(i=1)}^{\left(n\right)}{({HR}_{i}-{HR}_{mean})}^{2}} \text{C}\text{V}=\left(\frac{\text{S}\text{D}}{{\text{H}\text{R}}_{\text{m}\text{e}\text{a}\text{n}}}\right)\text{*}100.$$

### Outcome assessment

Follow-up was conducted by the trained neurologists who were blinded to the baseline information of patients by telephone or face-to-face visit. The primary outcome was all-cause mortality at 3-month. Additional outcomes included: change in modified Rankin Scale (mRS) score at 14-day and 3-month, respectively; the 14-day mortality. Death was confirmed by the death certificate from either the local citizen registry or the hospital where the patient was treated.

### Statistical analysis

Continuous variables were appropriately expressed as means with standard deviation (SD) or medians with interquartile range (IQR) and were analyzed by the student *t* test or Mann–Whitney *U* test according to their normality of distribution. Categorical variables were presented as proportions and analyzed by the χ2 or Fisher exact tests.

To standardize the analyses, we divided all measures of heart rate into tertiles with the lowest tertile as reference. The crude cumulative risks of 3-month mortality for each tertile group were shown in a Kaplan–Meier curve and compared using the log-rank test. Cox proportional hazards regression was used to estimate the risk of all-cause mortality. Hazard ratios (HRs) and 95% confidence intervals (CIs) were calculated for each group. Two multivariable regression models were used to analyze each clinical outcome. In model 1, we adjusted for age and sex. In model 2, we further adjusted for age, sex, medical history of hypertension, mTICI score and ASITN/SIR grading. To explore the effect of mean heart rate on functional outcome in the tertiles of heart rate SD and CV, we graphed the predicted probabilities of the individual values of the mRS from ordinal logistic regression. The effects of heart rate measures on mRS shift were analyzed using crude and multivariable ordinal logistic regression model adjusted for the same variables as for the mortality analyses. Two-sided *P* values of 0.05 were considered statistically significant. All statistics were conducted with SAS 9.4 software (SAS Institute Inc., Cary, NC). Figures were drawn by R software (R Development Core Team 2014, www.r-project.org).

## Results

A total of 191 LVO patients were finally included after excluding 14 patients for incomplete data on 72 h heart rate or baseline information and 9 for missing 3-month follow up. The baseline characteristics of the patients are shown in Table 1. Their mean age was 65.5 ± 14.2 years old and 112 (58.6%) patients were male. Symptomatic intracranial hemorrhage with 72 h occurred in 24 patients (12.6%). The median baseline NIHSS score was 16 (IQR, 13–20). During the 3-month follow-up, 51 patients (26.7%) died. Deceased patients were more likely to be elderly, had a history of hypertension, higher baseline NIHSS score, poor collaterals, less vessel recanalization after MT therapy, and significantly elevated mean heart rate, heart rate SD and heart rate CV.

**Table 1:** Baseline characteristics of mechanical thrombectomy treated acute ischemic stroke patients

	Entire Cohort (*n* = 191)	Alive *N* = 140 (73.3%)	Death *N* = 51 (26.7%)	*P* Value
Age, y; mean (SD)	65.5 (14.2)	63.4 (14.7)	71.1 (11.3)	< 0.001
Male sex, n (%)	112 (58.6)	86 (61.4)	26 (50.9)	0.20
History of hypertension, n (%)	129 (67.5)	86 (61.4)	43 (84.3)	0.003
History of diabetes mellitus, n (%)	31 (16.2)	19 (13.5)	12 (23.5)	0.10
History of atrial fibrillation, n (%)	81 (42.4)	61 (43.5)	20 (39.2)	0.59
History of prior stroke, n (%)	29 (15.1)	21 (15.0)	8 (15.6)	0.91
Current Smoking, n (%)	63 (32.9)	48 (34.2)	15 (29.4)	0.53
Current drinking, n (%)	42 (21.9)	33 (23.5)	9 (17.6)	0.38
Heart rate lowering treatment during the first 72 h, n (%)	36 (18.9)	27 (19.3)	9 (17.7)	0.80
Admission SBP, mean (SD)	146.7 (22.4)	144.6 (20.7)	152.6 (26.2)	0.07
Admission DBP, mean (SD)	85.3 (16.2)	84.2 (15.0)	88.6 (18.8)	0.12
Mean SBP 72 h after MT, mean (SD)	131.6 (16.8)	130 (14.5)	136 (21.3)	0.09
Admission NIHSS score, median (IQR)	16 (13–20)	15 (12–18)	21 (17–28)	< 0.001
Onset-to-revascularization time, median (IQR)	325 (261–435)	327 (260–441)	319 (263–414)	0.46
Low density lipoprotein, median (IQR)	2.71 (2.07–3.35)	2.70 (2.05–3.22)	2.96 (2.24–3.79)	0.17
High density lipoprotein, median (IQR)	1.17 (0.98–1.36)	1.17 (0.97–1.35)	1.22 (1.05–1.53)	0.17
Nt-pro BNP, median (IQR)	756 (170–1920)	728 (153–1828)	1057 (157–3235)	0.56
TOAST classification, n (%)				0.72
Large-artery atherosclerosis	81 (42.4)	57 (40.7)	24 (47.0)	
Cardioembolism	99 (51.8)	75 (53.5)	24 (47.0)	
Other determined or undetermined etiology	11 (5.7)	8 (5.7)	3 (5.8)	
Site of vessel occlusion, n (%)				0.05
ICA with or without MCA/ACA	20 (10.4)	12 (8.5)	8 (15.6)	
Isolated MCA or ACA	150 (78.5)	116 (82.8)	34 (66.6)	
Vertebrobasilar or other location	21 (10.9)	12 (8.5)	9 (17.6)	
tPA administered, n (%)	67 (35.1)	52 (37.1)	15 (29.4)	0.318
ASITN/SIR 2/3, n (%)	122 (64.6)	103 (74.6)	19 (37.3)	< 0.001
mTICI 2b/3, n (%)	154 (81.1)	119 (85.6)	35 (68.6)	0.008
Mean heart rate, median, mean (SD)	81.9 (15.5)	78.9 (13.1)	90.2 (18.5)	0.002
Heart rate SD, mean (SD)	12.2 (8.7)	10.9 (8.4)	15.7 (8.6)	0.02
Heart rate CV, mean (SD)	14.7 (9.3)	13.7 (9.3)	17.4 (8.9)	0.03
Symptomatic intracranial hemorrhage, n (%)	24 (12.6)	9 (6.4)	15 (29.4)	< 0.001

ACA indicates anterior cerebral artery; ASITN/SIR, the American Society of Interventional and Therapeutic Neuroradiology/Society of Interventional Radiology; DBP, diastolic blood pressure; heart rate SD, heart rate standard deviation; heart rate CV, heart rate coefficient of variation; ICA, internal carotid artery; NIHSS, National Institutes of Health Stroke Scale; MCA, middle cerebral artery; mTICI, modified Thrombolysis in Cerebral Ischemia; SBP, systolic blood pressure; TOAST, the Trial of Org 10,712 in Acute Stroke

### Association of heart rate measures with the all-cause mortality

**Fig. 1:**
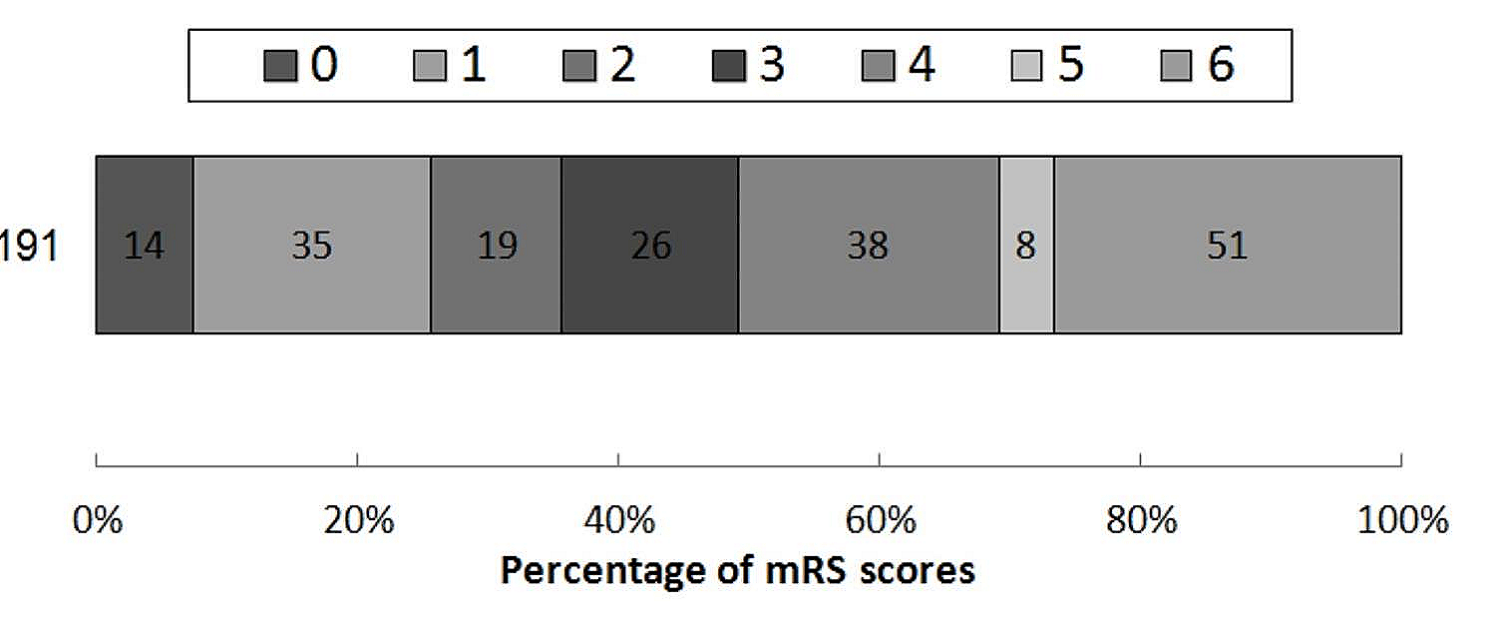
Distribution of mRS score at 90-d

**Fig. 2:**
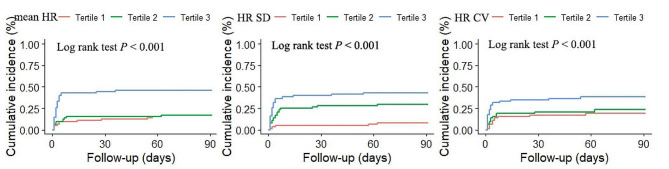
The association of BP values and mortality

Distribution of mRS score at 90-d are shown in Fig. 1. Kaplan-Meier survival curves were performed to estimate the association of mean heart rate, heart rate SD, heart rate CV and all-cause mortality in Fig. 2. Patients in the highest tertile of all heart rate measures had the highest cumulative risks of 3-month mortality (Table 2). In the crude COX model, there was a significant relationship between all heart rate measures and all-cause mortality. However, after adjustment for age, sex, hypertension, mTICI score and ASITN/SIR grading, only the highest tertile of heart rate SD had an increased risk of mortality (adjusted hazard ratio [aHR] with 95% CI: 4.62 [1.70-12.52], *P* = 0.003). The association between the highest tertile in mean heart rate or heart rate CV and mortality was nearly statistically significance (95% CI: 2.03 [0.99–4.16] and 2.02 [0.98–4.18], respectively). A linear relationship was observed between mean heart rate and heart rate SD with the mortality (*P* for trend: mean heart rate = 0.03, heart rate SD = 0.002, respectively), but not for the heart rate CV (*P* for trend = 0.05). Furthermore, when 72-hr mean heart rate increased by 10-bpm, the risk of mortality increased by 29% (aHR:1.29, 95% CI 1.09–1.51, *P* = 0.003). Meanwhile, when heart rate SD and heart rate CV increased by 5-unit, patients in the highest tertile increased their risk of mortality by 19% and 14% (aHR:1.19, 95% CI 1.07–1.32, *P* = 0.001; aHR:1.14, 95% CI 1.03–1.27, *P* = 0.01), respectively. We did not we did not find statistical association between heart rate and BP (*P* = 0.8194) in Figure I in the online Data Supplement.

**Table 2:** The association between heart rate measures and 3-month all-cause mortality in MT-treated acute ischemic stroke patients

Heart rate measures	*N*	Events, *n* (%)	Unadjusted			Model 1*			Model 2†		
			HR (95% Cl)	*P* value	*P* for trend	HR (95% Cl)	*P* value	*P* for trend	HR (95% Cl)	*P* value	*P* for trend
72-hr mean heart rate					< 0.001			< 0.001			0.03
Tertile 1 (< 73.6 bpm)	64	11 (17.18)	Reference			Reference			Reference		
Tertile2 (73.6–86.7 bpm)	64	11 (17.18)	1.02 (0.44–2.36)	0.96		1.08 (0.46–2.49)	0.86		0.85 (0.36–1.97)	0.70	
Tertile3 (> 86.7 bpm)	63	29 (46.03)	3.32 (1.66–6.66)	< 0.001		3.20 (1.59–6.46)	0.001		2.03 (0.99–4.16)	0.05	
per 10 bpm increase			1.44 (1.24–1.67)	< 0.001		1.42 (1.23–1.65)	< 0.001		1.29 (1.09–1.51)	0.003	
72-hr heart rate SD					< 0.001			< 0.001			0.002
Tertile 1 (< 8.3 unit)	64	5 (7.81)	Reference			Reference			Reference		
Tertile2 (8.3–12.1 unit)	64	19 (29.69)	4.23 (1.58–11.32)	0.004		4.13 (1.54–11.07)	0.005		2.91 (1.06–8.01)	0.04	
Tertile 3 (> 12.1 unit)	63	27 (42.86)	6.90(2.65–17.93)	< 0.001		6.87 (2.63–17.92)	< 0.001		4.62(1.70-12.52)	0.003	
per 5-unit increase			1.14 (1.06–1.23)	< 0.001		1.14 (1.05–1.23)	0.001		1.19 (1.07–1.32)	0.001	
72-hr heart rate CV					0.011			0.011			0.05
Tertile 1 (<10.8 unit)	64	12 (18.75)	Reference			Reference			Reference		
Tertile 2 (10.8–14.5 unit)	64	15 (23.44)	1.29 (0.61–2.76)	0.51		1.29 (0.60–2.75)	0.51		1.20 (0.56–2.56)	0.65	
Tertile 3 (>14.5 unit)	63	24 (38.10)	2.38 (1.19–4.76)	0.01		2.40 (1.20–4.80)	0.01		2.02 (0.98–4.18)	0.06	
per 5-unit increase			1.10 (1.04–1.23)	0.01		1.10 (1.02–1.19)	0.02		1.14 (1.03–1.27)	0.01	

*Model 1: Adjusted for age, sex;

†Model 2: Adjusted age, sex, medical history of hypertension, admission NIHSS score, preprocedural collateral status (ASITN/SIR 2/3), and postprocedural recanalization (mTICI score 2b/3)

ASITN/SIR indicates the American Society of Interventional and Therapeutic Neuroradiology/Society of Interventional Radiology; bpm, beat per minute; CI: confidence interval; HR, hazard ratio; heart rate SD, heart rate standard deviation; heart rate CV, heart rate coefficient of variation; NIHSS, National Institutes of Health Stroke Scale; MT, mechanical thrombectomy; mTICI, modified Thrombolysis in Cerebral Ischemia

The association between all heart rate measures and 14-day all-cause mortality are shown in Table I in the online Data Supplement. After adjustment for above mentioned variables in Table 2, the highest tertile in heart rate SD and heart rate CV were associated with an increased risk of all-cause mortality (*P* = 0.01 and 0.009, respectively). Interestingly, a linear relationship was also observed between heart rate SD or heart rate CV and all-cause mortality (*P* for trend: heart rate SD = 0.002, heart rate CV = 0.005). Furthermore, Furthermore, increased heart rate SD and heart rate CV was associated with increased 14-day mortality (*P* < 0.001 and *P* = 0.002, respectively). However, the association between mean heart rate and 14-day mortality was not significant statically.

### Association of heart rate measures with the worse functional outcome

The association between heart rate measures and 3-month worsening of functional outcome are shown in Table 3. In the crude ordinal regression analysis, all heart rate measures in patients in the highest tertiles were significantly associated with the increased risk of worsening of functional outcome. However, after adjusting for the same potential variables in Table 2, mean heart rate and heart rate SD still showed similar results (adjusted odds ratio (aOR) with 95% CI: mean heart rate, 2.73 [1.36–5.46]; heart rate SD, 3.52 [1.76–7.03]), except for the heart rate CV (*P* = 0.05). Meanwhile, a linear relationship was observed between all heart rate measures and 3-month increased risk of worsening of functional outcome (*P* for trend: mean heart rate = 0.005, heart rate SD < 0.001, heart rate CV = 0.049). Furthermore, higher heart rate and HRV were also associated with increased worsening of functional outcome: aOR:1.35, 95% CI 1.11–1.64, *P* = 0.002 per 10 unit increase in heart rate, and aOR:1.27, 95% CI 1.05–1.53, *P* = 0.01 and aOR:1.19, 95% CI 1.02–1.40, *P* = 0.03, per 5-unit increase in heart rate SD and heart rate CV respectively.

**Table 3:** The association between heart rate measures and 3-month worse functional outcome in MT-treated acute ischemic stroke patients

Heart rate measures	Unadjusted			Model 1*			Model 2†			
	OR (95% Cl)	*P* value	*P* for trend	OR (95% Cl)	*P* value	*P* for trend	OR (95% Cl)	*P* value	*P* for trend	
72-hr mean heart rate			< 0.001			< 0.001			0.005	
Tertile 1 (< 73.6 bpm)	Reference			Reference			Reference			
Tertile2 (73.6–86.7 bpm)	1.42 (0.77–2.61)	0.26		1.48 (0.80–2.74)	0.22		1.17 (0.61–2.24)	0.63		
Tertile3 (> 86.7 bpm)	4.38 (2.30–8.37)	< 0.001		4.29 (2.23–8.26)	< 0.001		2.73 (1.36–5.46)	0.005		
per 10-bpm increase	1.59 (1.32–1.91)	< 0.001		1.56 (1.30–1.88)	< 0.001		1.35 (1.11–1.64)	0.002		
72-hr heart rate SD			< 0.001			< 0.001			< 0.001	
Tertile 1 (< 8.3 unit)	Reference			Reference			Reference			
Tertile2 (8.3–12.1 unit)	3.28 (1.74–6.17)	< 0.001		3.47 (1.83–6.56)	< 0.001		2.48 (1.26–4.85)	0.008		
Tertile 3 (> 12.1 unit)	4.86 (2.54–9.30)	< 0.001		5.30 (2.74–10.25)	< 0.001		3.52(1.76–7.03)	< 0.001		
per 5-unit increase	1.47 (1.19–1.82)	< 0.001		1.46 (1.18–1.81)	< 0.001		1.27 (1.05–1.53)	0.01		
72-hr heart rate CV			0.02			0.007			0.049	
Tertile 1 (< 10.8 unit)	Reference			Reference			Reference			
Tertile 2 (10.8–14.5 unit)	1.59 (0.86–2.94)	0.14		1.67 (0.90–3.11)	0.10		1.53 (0.81–2.90)	0.19		
Tertile 3 (> 14.5 unit)	2.11 (1.13–3.93)	0.02		2.36 (1.26–4.43)	0.008		1.94 (0.99–3.80)	0.05		
per 5-unit increase	1.25 (1.04–1.50)	0.02		1.25 (1.04–1.50)	0.02		1.19 (1.02–1.40)	0.03		

*Model 1: Adjusted for age, sex;

†Model 2: Adjusted age, sex, medical history of hypertension, admission NIHSS score, preprocedural collateral status (ASITN/SIR 2/3), and postprocedural recanalization (mTICI score 2b/3). ASITN/SIR indicates the American Society of Interventional and Therapeutic Neuroradiology/Society of Interventional Radiology; bpm, beat per minute; CI, confidence interval; heart rate SD, heart rate standard deviation; heart rate CV, heart rate coefficient of variation; HR, hazard ratio; MT, mechanical thrombectomy; mTICI, modified Thrombolysis in Cerebral Ischemia; NIHSS, National Institutes of Health Stroke Scale

The association between all heart rate measures and 14-day functional outcome are shown in Tables II in the online Data Supplement. There was a significant relationship between all heart rate measures and worsening of functional outcome both in the crude and multivariate-adjusted shift analysis (all *P* < 0.05). In addition, a linear relationship was observed between all heart rate measures and worsening of functional outcome (all *P* < 0.05). Similarly, increased 72-hr mean heart rate, heart rate SD and heart rate CV modeled as continuous variables were associated with worsening of functional outcome (all *P* < 0.05).

AF may have an impact on variations of heart rate. We conducted a subgroup analysis, and we found that heart rate value was associated with poor functional outcome in both patients with and without AF in Table III in the online Data Supplement.

## Discussion

In this prospective study of MT-treated AIS patients, heart rate measures, including mean heart rate, heart rate SD and CV, were associated with an increased risks of the 3-month all-cause mortality and worsening of functional outcome, especially in those with heart rate levels and variability expressed as continuous variables (mean heart rate increased by 10-bpm, heart rate SD and CV increased by 5-unit). Our study suggested that HRV and mean heart rate, indicating the overall adjustment to a stress response, may be useful for predicting clinical outcomes in MT-treated AIS patients.

The imbalance between sympathetic and parasympathetic systems often occurs in the acute phase of stroke and represents an abnormal autonomic function in response to the severity of disease. The mean heart rate and HRV are the most frequently used approach to evaluate the cardiovascular autonomic regulatory system because it is a non-invasive and easily accessible tool. A large number of previous evidence demonstrated a significant association between admission resting heart rate and poor clinical outcomes [25–27]. However, these were only one-time random recording of heart rate and might not accurately reflect the heart rate at the onset of stroke. Therefore, continuous dynamic heart rate recording and subsequent analysis of the mean heart rate and HRV has more clinical significance. A few studies have shown that abnormal HRV was associated with the higher risk of mortality in the prognosis of coronary artery disease [12]. However, the impact of HRV was inconclusive in stroke studies. In a cohort study that included AIS patients with AF, the association between the mean heart rate and 1-year death was significant but not in heart rate CV [10]. One study that only included patients with the sinus rhythm and utilized different parameters of HRV showed reduced HRV was related to an unfavorable functional outcome [28]. The different time course of heart rate monitoring from stroke onset to the recording, the different measurements to assess HRV and the follow-up period might have different effect on the predictive value of heart rate levels and HRV. The impact of the mean heart rate or HRV on the outcome in LVO patients, especially those underwent MT therapy, has not been explored yet. Yang et al. reported that increased resting heart rate variability combined may increase the risk of all-cause mortality [29], which is similar to our results. Our study found that higher HRV is associated with poor outcome. This reason may be that all patients were routinely admitted to the intensive care unit for at least 72 h after MT and were required to be bedridden. Patients with severe cerebral infarction are more susceptible to external stimuli that cause heart rate fluctuations. Our study provided the evidence that both the mean heart rate and HRV (heart rate SD and heart rate CV) had a significant impact on the clinical outcomes, and could be potentially used as surrogate markers of autonomic dysfunction accounting for a poor outcome in MT-treated LVO stroke patients.

Several mechanisms possibly explain the association between HRV or mean heart rate and increased risk of mortality in patients. First, the disturbance of autonomic nervous system may attribute to the damage of central autonomic nervous system structures, such as central cortex and brain stem, which would affect the cardiac function or causing arrhythmias in clinical and experimental studies [30–33]. Second, incremental heart rate may reflect sympathetic overactivity. Third, the dysfunctional HRV and mean heart rate are responses to the acute stress disorders that may amplify subsequent adverse outcome [34].

To our knowledge, this is the first study that focused on the association between the prognostic factor of heart rate measures and the outcome of AIS patients treated with MT. There are several limitations in our study. First, it was a single-center, observational study with a small sample size. The generalization of our results to other clinical practice is uncertain. A multicenter, largescale cohort study is needed to validate our findings. Second, information on antiarrhythmic agents was not collected in this study. The antiarrhythmic agents might influence the heart rate measures. Third, the heart rate information was collected hourly. Further long-term continuous dynamic heart rate recording may be needed.

## Conclusions

Our study showed that higher mean heart rate and HRV were associated with the increased risk of all-cause mortality and worse functional outcome after MT therapy in AIS patients at 3-month visit. These findings suggested that higher heart rate and HRV may be a potential risk factors for worsening of prognosis in MT-treated AIS patients. Timely monitor and manage heart rate variation may be beneficial in these patients.

### Electronic supplementary material

Below is the link to the electronic supplementary material.

Supplementary Material 1

## Data Availability

The datasets generated and/or analysed during the current study are not publicly available due to the datasets are owned by the institution only but are available from the corresponding author on reasonable request.

## Abbreviations

AIS: acute ischemic stroke
ASITN/SIR: American Society of Interventional and Therapeutic Neuroradiology/Society of Interventional Radiology
Bpm: beat per minute
HRV: heart rate variability
LVO: large vessel occlusion
mRS: modified Rankin scale
MT: mechanical thrombectomy
mTICI: modified Thrombolysis in Cerebral Infarction
NIHSS: National Institutes of Health Stroke Scale
