# Supplementary material for: Effect of heart rate on poor outcome in stroke patients treated with intra-arterial thrombectomy
Source: BMC Neurol. 2024 May 21;24:164. doi: 10.1186/s12883-024-03662-8 (PMC11106893; doi:10.1186/s12883-024-03662-8)
Supplement: Supplementary file 1 — Supplementary Material 1 [file 12883_2024_3662_MOESM1_ESM.docx]

**Supplement Table I**: The association between heart rate measures and 14-day all-cause mortality in MT-treated AIS patients.

| Heart rate measures | N | Events, n(%) | Unadjusted | | | Model 1* | | | Model 2† | | |
| --- | --- | --- | --- | --- | --- | --- | --- | --- | --- | --- | --- |
|  |  |  | HR (95% Cl) | *P*  value | *P* for trend | HR (95% Cl) | *P*  value | *P* for trend | HR (95% Cl) | *P*  value | *P* for trend |
| 72-hr mean heart rate |  |  |  |  | 0.002 |  |  | 0.003 |  |  | 0.08 |
| Tertile 1 (<73.6 bpm) | 64 | 5 (7.81) | Reference |  |  | Reference |  |  | Reference |  |  |
| Tertile2 (73.6-86.7 bpm) | 64 | 7 (10.94) | 1.42 (0.45-4.49) | 0.55 |  | 1.44 (0.46- 4.56) | 0.53 |  | 1.06 (0.33- 3.38) | 0.93 |  |
| Tertile3 (> 86.7 bpm) | 63 | 39 (61.90) | 4.22 (1.57-11.39) | 0.005 |  | 4.05 (1.49-11.00) | 0.006 |  | 2.15 (0.78- 5.96) | 0.14 |  |
| per 10 bpm increase |  |  | 1.42 (1.17-1.72) | <0.001 |  | 1.40 (1.16-1.70) | <0.001 |  | 1.24 (0.99-1.54) | 0.060 |  |
| 72-hr heart rate SD |  |  |  |  | <0.001 |  |  | <0.001 |  |  | 0.002 |
| Tertile 1 (<8.3 unit) | 64 | 2 (3.13) | Reference |  |  | Reference |  |  | Reference |  |  |
| Tertile2 (8.3-12.1 unit) | 64 | 8 (12.50) | 4.26 (0.91-20.08) | 0.07 |  | 4.18 (0.89-19.68) | 0.07 |  | 2.80 (0.58-13.59) | 0.20 |  |
| Tertile 3 (> 12.1 unit) | 63 | 41 (65.08) | 11.83 (2.76-50.66) | <0.001 |  | 11.56 (2.69-49.65) | 0.001 |  | 6.89 (1.54-30.93) | 0.01 |  |
| per 5-unit increase |  |  | 1.17 (1.08-1.27) | <0.001 |  | 1.18 (1.08-1.29) | <0.001 |  | 1.28 (1.12-1.45) | <0.001 |  |
| 72-hr heart rate CV |  |  |  |  | 0.001 |  |  | 0.001 |  |  | 0.005 |
| Tertile 1 (<10.8 unit) | 64 | 4 (6.25) | Reference |  |  | Reference |  |  | Reference |  |  |
| Tertile 2 (10.8-14.5 unit) | 64 | 8 (12.50) | 2.06 (0.62-6.82) | 0.24 |  | 2.03 (0.61-6.75) | 0.25 |  | 2.04 (0.61-6.80) | 0.25 |  |
| Tertile 3 (> 14.5 unit) | 63 | 39 (61.90) | 5.11 (1.73-15.12) | 0.003 |  | 5.11 (1.73-15.12) | 0.003 |  | 4.43 (1.45-13.57) | 0.009 |  |
| per 5-unit increase |  |  | 1.13 (1.04-1.23) | 0.004 |  | 1.14 (1.04-1.24) | 0.004 |  | 1.22 (1.07-1.38) | 0.002 |  |

*Model 1: Adjusted for age, sex;

†Model 2: Adjusted age, sex, medical history of hypertension, Admission NIHSS score, preprocedural collateral status (ASITN/SIR 2/3), and postprocedural recanalization (mTICI score 2b/3).

MT indicates mechanical thrombectomy; HR, hazard ratio; bpm, beat per minute ; heart rate SD, heart rate standard deviation; heart rate CV, heart rate coefficient of variation; NIHSS, National Institutes of Health Stroke Scale; ASITN/SIR, the American Society of Interventional and Therapeutic Neuroradiology/Society of Interventional Radiology; mTICI, modified Thrombolysis in Cerebral Ischemia.

**Supplement Table II:** The association between heart rate measures and 14-day worse functional outcome in MT-treated AIS patients (Multivariable Ordinal Logistic Regression)

| Heart rate measures | Unadjusted | | | Model 1* | | | Model 2† | | |
| --- | --- | --- | --- | --- | --- | --- | --- | --- | --- |
|  | OR(95%Cl) | *P* value | *P* for trend | OR(95%Cl) | *P* value | *P* for trend | OR(95%Cl) | *P* value | *P* for trend |
| 72-hr mean heart rate |  |  | <0.001 |  |  | <0.001 |  |  | 0.008 |
| Tertile 1 (<73.6 bpm) | Reference |  |  | Reference |  |  | Reference |  |  |
| Tertile2 (73.6-86.7 bpm) | 1.45 (0.77- 2.73) | 0.25 |  | 1.49 (0.79-2.81) | 0.22 |  | 0.99 (0.51-1.91) | 0.97 |  |
| Tertile3 (> 86.7 bpm) | 4.87 (2.49- 9.53) | <0.001 |  | 4.52 (2.30-8.89) | <0.001 |  | 2.66 (1.31-5.41) | 0.007 |  |
| per 10 bpm increase | 1.55 (1.30-1.85) | <0.001 |  | 1.52 (1.27-1.82) | <0.001 |  | 1.31 (1.08-1.58) | 0.005 |  |
| 72-hr heart rate SD |  |  | <0.001 |  |  | <0.001 |  |  | <0.001 |
| Tertile 1 (<8.3 unit) | Reference |  |  | Reference |  |  | Reference |  |  |
| Tertile2 (8.3-12.1 unit) | 3.98 (2.05- 7.71) | <0.001 |  | 3.95 (2.03-7.66) | <0.001 |  | 3.06 (1.52-6.18) | 0.002 |  |
| Tertile 3 (> 12.1 unit) | 5.71 (2.89- 11.25) | <0.001 |  | 5.76 (2.91-11.42) | <0.001 |  | 4.42 (2.15-9.12) | <0.001 |  |
| per 5-unit increase | 1.57 (1.26-1.94) | <0.001 |  | 1.55 (1.25-1.92) | <0.001 |  | 1.39 (1.15-1.69) | <0.001 |  |
| 72-hr heart rate CV |  |  | 0.004 |  |  | 0.002 |  |  | 0.004 |
| Tertile 1 (<10.8 unit) | Reference |  |  | Reference |  |  | Reference |  |  |
| Tertile 2 (10.8-14.5 unit) | 1.51 (0.80- 2.82) | 0.20 |  | 1.54 (0.82-2.90) | 0.18 |  | 1.49 (0.78-2.85) | 0.23 |  |
| Tertile 3 (> 14.5 unit) | 2.58 (1.36- 4.91) | 0.004 |  | 2.79 (1.46-5.32) | 0.002 |  | 2.80 (1.41-5.55) | 0.003 |  |
| per 5-unit increase | 1.33 (1.10-1.61) | 0.004 |  | 1.32 (1.09-1.59) | 0.004 |  | 1.28 (1.09-1.51) | 0.002 |  |

*Model 1: Adjusted for age, sex;

†Model 2: Adjusted age, sex, medical history of hypertension, Admission NIHSS score, preprocedural collateral status (ASITN/SIR 2/3), and postprocedural recanalization (mTICI score 2b/3).

MT indicates mechanical thrombectomy; HR, hazard ratio; bpm, beat per minute ; heart rate SD, heart rate standard deviation; heart rate CV, heart rate coefficient of variation; NIHSS, National Institutes of Health Stroke Scale; ASITN/SIR, the American Society of Interventional and Therapeutic Neuroradiology/Society of Interventional Radiology; mTICI, modified Thrombolysis in Cerebral Ischemia.

**Supplement Table III:** The association between HR measures and 3-month worse outcome in MT-treated acute ischemic stroke patients. (Multivariable Ordinal Logistic Regression)

| **Variable** | **OR (95%CI)** | ***P*** | | |
| --- | --- | --- | --- | --- |
| Non AF |  | |  |  |
| Mean HR per 10 bpm | 1.10 (1.02-1.17) | | 0.007 |  |
| HRCV per 5 unit | 1.70 (1.15-2.73) | | 0.015 |  |
| AF |  | |  |  |
| Mean HR per 10 bpm | 1.87 (1.31-2.85) | | 0.002 |  |
| HRCV per 5 unit | 1.53 (1.07-2.01) | | 0.031 |  |

**Supplement Figure I:** The association between mean SBP and mean HR in MT-treated AIS patients (Linear Regression)


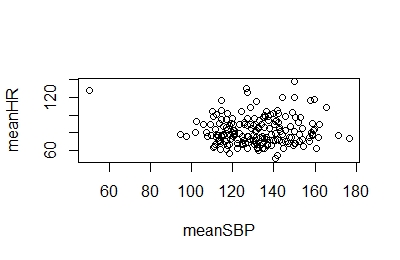
*P* = 0.819
